# From the Literature on Mining to Computational Verification: A Review of the Anti-Radiation Mechanisms of Sulfur Compounds in the Seeds of *Lepidium apetalum* Willd and *Descurainia sophia* (L.) Webb ex Prantl

**DOI:** 10.3390/ijms27041847

**Published:** 2026-02-14

**Authors:** Zhenzhen Wei, Yujie Wang, Yuan Lu, Chao Yang, Ke Wen, Chunyan Feng, Jianfeng Yi, Qian Liu

**Affiliations:** 1 Integrated Chinese and Western Medicine Institute for Children Health and Drug Innovation, Jiangxi University of Chinese Medicine, Nanchang 330004, China; 2 Institute for Advanced Study, Jiangxi University of Chinese Medicine, Nanchang 330004, China; 3 Research Center for the Differentiation and Development of Traditional Chinese Medicine Basic Theory, Jiangxi University of Chinese Medicine, Nanchang 330004, China

**Keywords:** sulfur-containing radiation protection agents, toxic side effects, radiation damage, sulfur compounds, PI3K/AKT

## Abstract

Compounds containing sulfur are the primary components of anti-radiation drugs and represent a key focus in the innovative design and discovery of pharmaceuticals. The adverse effects of synthetic sulfur-containing radiation protective agents are significant concerns that cannot be overlooked. It is imperative to identify natural sulfur compounds that exhibit low toxicity and high efficacy as radiation protection agents. Cruciferous plants demonstrate notable resistance to ionizing radiation. The literature review revealed that *Lepidii semen* and *Descurainiae semen*, both of which are rich in sulfur compounds and the PI3K/AKT signaling pathway regulates radiation-induced oxidative stress, inflammation, and apoptosis. We speculate that the sulfur compounds of the *Lepidii semen* and *Descurainiae semen* may exert radiation protection by regulating the PI3K/AKT signaling pathway, and this hypothesis was supported by molecular docking analysis. The sulfur compounds (glucotropaeolin, gluconapin, glucoiberverin, glucocappasalin, tropeolin, etc.) demonstrate greater potential.

## 1. Introduction

In recent years, the rapid advancement of nuclear science and the widespread utilization of nuclear technology have brought convenience to humanity, but also increased exposure to ionizing radiation. Ionizing radiation can directly attack the body’s DNA, proteins, lipids, and other biological macromolecules, and can also ionize water molecules to produce a large number of free radicals, which indirectly damage cells and, thus, harm the circulatory, immune, reproductive, and nervous systems [1,2,3,4,5]. Research and the development of radioprotective and radiotherapeutic agents are particularly important. Sulfur-containing compounds are the primary components of anti-radiation drugs and represent a key focus in the innovative design and discovery of pharmaceuticals [6,7]. The adverse effects of synthetic sulfur-containing radiation-protective agents are significant concerns that cannot be overlooked.

**Figure 1:**
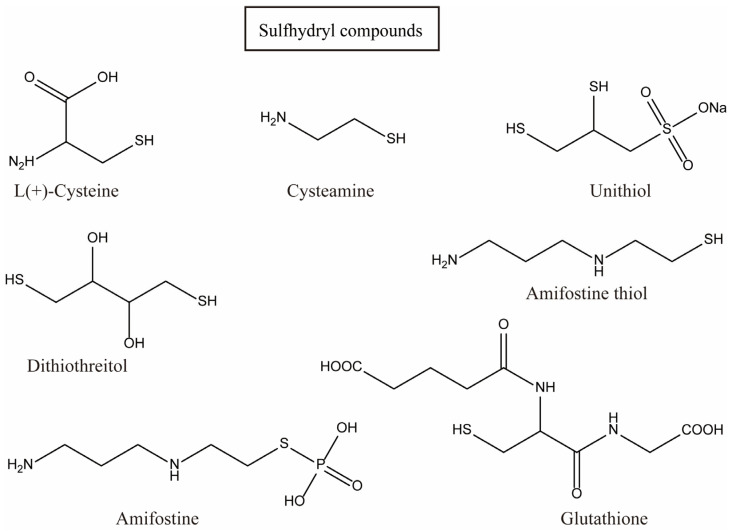
Sulfhydryl compounds with anti-radiation activity.

**Figure 2:**
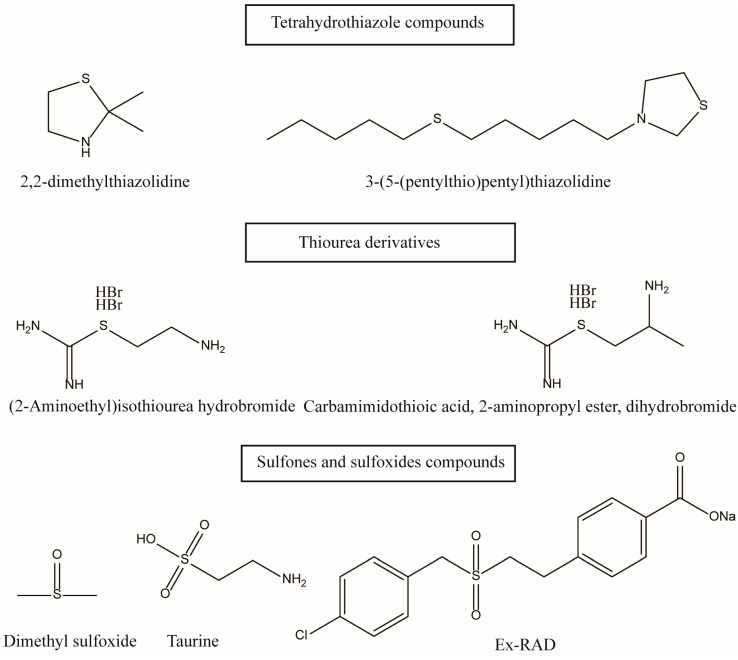
Disulfide compounds, tetrahydrothiazole compounds, thiourea derivatives and sulfone and sulfoxide compounds with anti-radiation activity.

**Figure 3:**
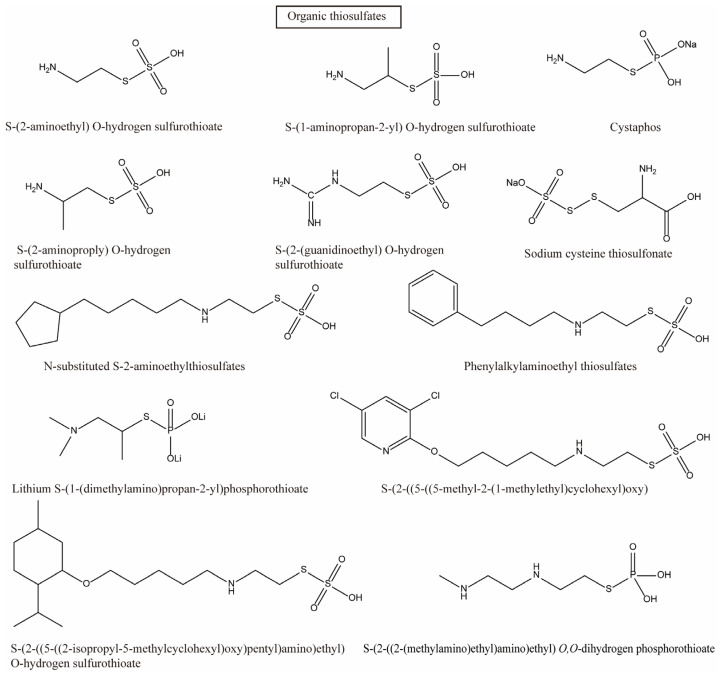
Organic thiosulfates with anti-radiation activity.

Early scientists found many compounds with anti-radiation activity in sulfur-containing compounds (Figure 1, Figure 2 and Figure 3), such as sulfhydryl compounds, disulfide compounds, organic thiosulfate, tetrahydrothiazole compounds, thiourea derivatives, and sulfone and sulfoxide compounds; for detailed information, please refer to Table 1.

Despite their potent capacity to scavenge free radicals and mitigate radiation-induced cellular damage, sulfur-containing compounds as radioprotective agents have been a significant focus of research in this field. However, more effective radioprotective agents are often accompanied by significant side effects, such as nausea, vomiting, hypotension, and weakness, as seen with amifostine, Ex-RAD, and Prc-210 [36,37,38,39]. While these adverse reactions are generally not life-threatening, they can significantly impact users’ quality of life, which significantly limits the clinical use of these drugs.

Natural products characterized by high biological activity and low toxicity have garnered significant attention in recent years. Identifying antiradiation compounds from natural products is crucial for the development of radiation protective agents. However, the protective effects of natural sulfur compounds against radiation have not yet been systematically investigated.

A substantial body of prior research has demonstrated that cruciferous plants are a part of the plant class known for their robust resistance to radiation. The Shanghai Institute of Plant Biology, Chinese Academy of Sciences, has discovered that the radiation resistance of cruciferous plants is attributed to the presence of natural radiation protection substances in their bodies [40]. Many of these substances possess potent antioxidant and anti-inflammatory properties, which can mitigate radiation-induced damage by attenuating oxidative stress and inflammatory responses.

However, a significant gap exists in the systematic review of natural sulfur compounds, particularly from specific medicinal plants, for radioprotection. While the radioprotective potential of common cruciferous vegetables like broccoli and cabbage is increasingly recognized, the focus on their seeds—especially those with a history of medicinal use—remains underexplored. This review aims to fill this gap by critically evaluating the radioprotective potential of sulfur compounds derived specifically from *Lepidii semen* (the seed of *Lepidium apetalum* Willd.) and *Descurainiae semen* (the seed of *Descurainia sophia* (L.) Webb ex Prantl).

This study constitutes a structured narrative review. A systematic search was performed to identify the relevant literature, which was then integrated narratively. Since the goal was to scope and synthesize a wide range of evidence rather than to test a precise clinical hypothesis, we did not follow the PRISMA checklist. This choice of methodology is explicitly declared and rationalized in the following literature search strategy.

The selection of these two seeds is based on several compelling reasons. First, as established repositories of diverse sulfur-containing phytochemicals predominantly concentrated in cruciferous seeds [41], they possess compound profiles that may differ from other plant parts or species. Second, they have a longstanding application in Traditional Chinese Medicine for treating conditions such as asthma, edema, and dysuria [42,43], which implies inherent bioactivities relevant to cellular protection and inflammation modulation—key aspects of radiation injury. This ethnopharmacological rationale provides a strong foundation for investigating their mechanistic role in radioprotection. This review will systematically consolidate the evidence linking the unique sulfur compounds in these seeds to radioprotective mechanisms. Furthermore, to clearly delineate their unique position, we will include a comparative analysis contrasting *Lepidii semen* and *Descurainiae semen* with other well-studied cruciferous plants, highlighting why these specific seeds warrant dedicated research efforts.

## 2. Literature Search Strategy

To ensure a comprehensive and systematic collection of relevant literature and compound data, a well-defined search strategy was employed. The methodology is detailed as follows:

(1) Data Sources and Search Strategy for Sulfur Compounds. The sulfur compounds found in *Lepidii semen* and *Descurainiae semen* were primarily retrieved from specialized databases, including TCMSP (https://www.tcmsp-e.com/ (accessed on 28 October 2024)), PubMed (https://pubmed.ncbi.nlm.nih.gov/), and SciFinder (https://scifinder.cas.org/) [44]. These platforms were queried using the seed names (“*Lepidii semen*”, “*Lepidium apetalum*”, “*Descurainiae semen*”, “*Descurainia sophia*”) as primary keywords to identify their known chemical constituents, with a focus on sulfur-containing compounds.
(2) Systematic Literature Review for Radioprotective Effects. To gather literature on the radioprotective effects and mechanisms, a systematic search was conducted across PubMed, Web of Science, and China National Knowledge Infrastructure (CNKI). The search was designed to cover publications from the inception of the respective databases through the end of March 2025 to ensure comprehensiveness. The search strategy utilized a combination of keywords and Boolean operators (AND, OR) to maximize relevance: (Radioprotect OR “radiation protection” OR “anti-radiation” OR “radiotherapy side effects”) AND (Sulfur compound OR “glucosinolates” OR “isothiocyanates” OR “thiocyanate”) AND (“*Lepidii semen*” OR “*Lepidium apetalum*” OR “*Descurainiae semen*” OR “*Descurainia sophia*” OR “*Tinglizi*”) AND (Pathway OR mechanism OR “PI3K/AKT” OR “Nrf2” OR “NF-κB” OR antioxidant OR anti-inflammatory).
(3) Inclusion and Exclusion Criteria. Inclusion Criteria: Studies published in peer-reviewed journals; Studies that investigated the chemical constituents, specifically sulfur-containing compounds, of *Lepidii semen* or *Descurainiae semen*; Studies that evaluated the radioprotective, antioxidant, or anti-inflammatory effects of these seeds or their isolated compounds, either in vitro or in vivo; Studies that explored the underlying molecular mechanisms. Exclusion Criteria: Non-peer-reviewed publications, conference abstracts, patents, or theses (unless providing critical unique data); Studies not focused on the radioprotective or direct pharmacological properties relevant to radiation damage.
(4) Study Selection and Data Extraction. The literature search and screening process were conducted by two independent authors to minimize bias. Initially, titles and abstracts were screened to exclude irrelevant studies. Subsequently, the full texts of the remaining articles were reviewed against the inclusion and exclusion criteria. Discrepancies were resolved through discussion until a consensus was reached. Key data from the included studies, such as the type of sulfur compound, observed biological effects, and proposed mechanisms, were extracted and synthesized in this review.

## 3. Exploring Potential Mechanisms

### 3.1. The PI3K/AKT Signaling Pathway: A Key Mechanistic Framework for Radioprotection

#### 3.1.1. Pathway Overview and Relevance to Radiation Response

The phosphatidylinositol 3-kinase (PI3K)/protein kinase B (PKB or Akt) signaling pathway is ubiquitous in various cell types, playing a critical role in cell growth, proliferation, and differentiation. This pathway is intrinsically linked to the fundamental cellular processes of oxidative stress, inflammation, and apoptosis (Pan et al., 2023) [45]. PI3K, composed of a catalytic subunit p110 and a regulatory subunit p85, serves as a crucial coordinating factor for intracellular signaling in response to extracellular stimuli. Its phosphorylation leads to the generation of phosphatidylinositol 3,4,5-triphosphate (PIP3), which in turn recruits and phosphorylates Akt, the key kinase in the pathway, to modulate various physiological activities, including oxidative stress, inflammation, apoptosis, and growth [46].

Substantial evidence demonstrates that the PI3K/AKT pathway is activated following radiation-induced injury and plays a critical role in promoting cell survival by modulating these damaging processes [47,48,49,50]. This activation promotes cell survival following radiation exposure [51,52]. Key mechanisms include the facilitation of DNA repair, where elevated levels of Akt and mTOR phosphorylation promote the recruitment of DNA-PKCs and MRE11 to double-strand break sites for DNA repair and enhanced cell survival [53,54]. Notably, irradiation significantly increased apoptosis in the ileal crypts of Akt knockout mice, underscoring the correlation between PI3K/AKT pathway activation and resistance to ionizing radiation [55]. Furthermore, this pathway is implicated in specific radiation-induced injuries, such as myocardial damage via the VEGFR2/PI3K/AKT axis [56]. Specifically, radioprotective agents may exert their effects through this pathway via several mechanisms: mitigating oxidative stress by activating the PI3K/AKT-Nrf2/HO-1 axis, thereby reducing intracellular ROS accumulation [48,57,58]; regulating the inflammatory response through the PI3K/AKT/NF-κB pathway, which controls the release of factors like TNF-*α*, IL-1*β*, IL-6, and COX-2 [48,59]; and inhibiting cellular apoptosis via the PI3K/AKT/mTOR signaling pathway [60,61].

The Nrf2/Keap1 pathway is the core controller of the cellular antioxidant stress defense system [62]. It is strictly inhibited by Keap1 under physiological conditions and rapidly activated under stress, restoring and maintaining intracellular homeostasis by coordinating the expression of a vast gene network [63]. This pathway is an important target for the prevention and treatment of numerous oxidative stress-related diseases. Overactivated Nrf2 provides strong protection for cells, enabling them to resist high levels of ROS and chemotherapy/radiotherapy [64,65]. Sulforaphane, which is abundant in cruciferous vegetables such as broccoli, is one of the most famous natural activators of Nrf2 [66,67].

#### 3.1.2. Mechanistic Link to Radioprotective Phenotypes

**Figure 4:**
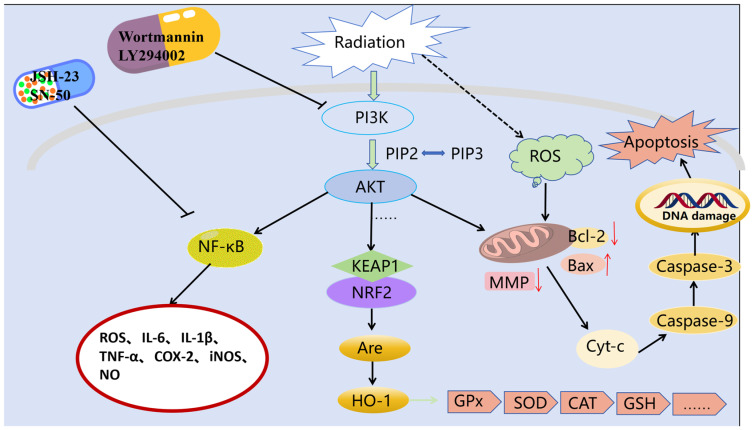
Regulation of PI3K/AKT Pathway to Mitigate Radiation-Induced Oxidative Stress, Inflammation, and Apoptosis: A Schematic Overview. Note: This schematic diagram illustrates the central role of the PI3K/AKT signaling pathway in counteracting key adverse effects of ionizing radiation (IR) exposure: oxidative stress, inflammation, and apoptosis. (→: Promotes; —: Inhibits; ↓: Decreased expression level; ↑: Increased expression level).

The radioprotective effect mediated by PI3K/AKT activation can be understood through its precise regulation of downstream effectors, which aligns with the modern shift in antiradiation drug development from solely scavenging free radicals [50] towards understanding the cascade reaction of cell repair post-radiation. Anti-radiation agents can exert their effects by modulating the expression of key pathway factors, inhibiting oxidative stress response, inflammatory factor release and cell apoptosis [68]. It primarily exerts its effects through the following mechanisms (Figure 4):

(1) Alleviating oxidative stress: By activating the PI3K/AKT-Nrf2/HO-1 pathway, it inhibits the accumulation of intracellular reactive oxygen species (ROS), thereby mitigating oxidative stress damage [69,70,71].
(2) Modulating inflammatory responses: Activation of the PI3K/AKT signaling pathway upregulates NF-κB, which in turn promotes the expression and release of inflammatory factors such as TNF-α, IL-1β, IL-6, IL-10, NO, iNOS, and COX-2 [72,73].
(3) Inhibiting apoptosis: The PI3K/AKT/mTOR pathway and its downstream effectors are involved in regulating the apoptotic process, exerting an inhibitory effect on it [74].

### 3.2. The Chemical Composition and Pharmacological Activity of Descurainiae Semen and Lepidii Semen

To date, a total of 87 chemical constituents have been isolated from *Descurainiae semen* and *Lepidii semen,* including glucosinolates, isothiocyanates and sinapate derivatives, flavonoids, cardiac glycosides, phenylpropanoids, organic acids, other compounds, as well as fatty oils, as listed in Table 2. Overall, research on the chemical constituents of *Descurainiae semen* has been more extensive than that on *Lepidii semen*, which is likely attributable to the fact that *Descurainiae semen* represents the mainstream commercial product in the market [43].

Pharmacological studies have shown that both *Descurainiae semen* and *Lepidii semen* have cardiovascular function-improving effects and cytotoxic effects [75]. *Lepidii semen* also exhibits antitussive effects [42]. *Descurainiae semen* has diuretic, lipid-regulating, and central nervous system-modulating effects [43]. In addition, *Descurainiae semen* oil demonstrated positive results in mouse antioxidant tests, including the determination of malondialdehyde content, superoxide dismutase activity, and fruit fly survival experiments, indicating its potential anti-aging activity [76]. Moreover, *Lepidii semen* oil also exhibits antioxidant activity [77]. *Lepidium apetalum* Willd. extracts significantly attenuated airway mucus hypersecretion, inflammatory cell infiltration—including eosinophils—and eosinophil activation in preclinical models. Moreover, these extracts suppressed the expression of type 2 cytokines (e.g., IL-4, IL-5, and IL-13) and inhibited both the differentiation and functional activation of T helper 2 (Th2) cells [42]. Sulfur-containing glycosides isolated from *L. apetalum* Willd. demonstrated potent anti-inflammatory activity, effectively mitigating hypertonicity-induced inflammatory responses in renal epithelial cells [49,78]. Benzyl isothiocyanate—a major hydrolysis product of glucosinolates in this plant—exerts multi-target pharmacological effects, including anti-inflammatory, anticancer, and antioxidant activities, partly through modulation of the PI3K/AKT signaling pathway [79,80,81]. Collectively, the glucosinolates present in *L. apetalum* Willd. seeds—and their enzymatically derived isothiocyanates—are considered the primary bioactive constituents responsible for their antioxidant, anti-inflammatory, and radioprotective properties.

However, there have been no studies on the radioprotective effects of *Descurainiae semen* and *Lepidii semen* to date. Based on the analysis of their chemical constituents and biological activities, we hypothesize that both *Descurainiae semen* and *Lepidii semen* likely possess significant radioprotective activity, with organosulfur compounds primarily serving as the material basis for this effect.

**Table 2:** Chemical Composition of *Descurainiae semen* and *Lepidii semen*.

No.	Chemical Composition	Classification of Compounds	Plant Source	References
1	descurainoside	Organic sulfur compounds	1	[82]
2	raphanuside B		1	[83]
3	gluconapin		1	[84]
4	glucoiberverin		1	[84]
5	glucotropaeolin		1	[84]
6	glucocappasalin		1	[84]
7	1-(methylsulfinyl)hexan-3-ol		1	[84]
8	diallyl disulfide		1	[85]
9	apetalumosides D		1	[47]
10	raphanuside C		1	[83]
11	lepidiumside F		1	[84]
12	raphanuside D		1	[83]
13	myronate		2	[85]
14	sinalbin		1	[85]
15	lepidiumflavonosides A		2	[48]
16	lepidiumflavonosides B		2	[48]
17	1-thio-β-d-glucopyranosyl(1→1)-1-thio-α-d-glucopyranoside		2	[47]
18	TgSSTg		2	[47]
19	cis-desulfoglucotropaeolin (cis-DG)		2	[49]
20	trans-desulfoglucotropaeolin (trans-DG)		2	[49]
21	(2-isothiocyanatoethyl)benzene		1, 2	TCMSP
22	tropeolin		1, 2	TCMSP
23	butenylisothiocyanate		1, 2	TCMSP
24	mustard oil		1, 2	TCMSP
25	urogran		1, 2	TCMSP
26	phenylmethanethiol		1, 2	TCMSP
27	kaempferol	Flavonoid compounds	1	[86,87]
28	isorhamnetin		1	[88]
29	quercetin		1, 2	[87,89]
30	isorhamnetin-3-O-β-D-glucopyranoside		1, 2	[88,89]
31	quercetin-3-O-β-D-glucopyranoside		1, 2	[88,89]
32	descurainin A		1	[90,91]
33	quercetin-3-O-β-D-glucopyranosyl-7-O-β-gentiobioside		1	[92]
34	kaempferol-3-O-β-D-glucopyranosyl-7-O-β-gentiobioside		1	[92]
35	isorhamnetin-3-O-β-D-glucopyranosyl-7-O-β-gentiobioside		1	[92]
36	quercetin-7-O-β-gentiobioside		1	[87,92]
37	kaempferol-7-O-β-gentiobioside		1	[92]
38	isorhamnetin-7-O-β-gentiobioside		1	[92]
39	quercetin-3,7-di-O-β-D-glucopyranoside		1	[92]
40	kaempferol-3,7-di-O-β-D-glucopyranoside		1	[92]
41	isorhamnetin-3,7-di-O-β-D-glucopyranoside		1	[92]
42	kaempferol-3-O-β-D-glucopyranosyl-7-O-[(2-O-trans-sinapoyl)-β-D-glucopyranosyl(1→6))-β-D-glucopyranoside		1	[92]
43	drabanemoroside		1	[88]
44	quercetin-3-O-α-L-rhamnopyranosyl-(1→2)-α-L-arabinopyranose		1	[88]
45	quercetin-3-O-β-D-[2-O-(6-O-sinapoyl)-β-D-glucopyranosyl)-glucopyranoside		2	[89]
46	isorhamnetin-3-O-β-D-[2-O-(6-O-sinapoyl)-β-D-glucopyranosyl)-glucopyranoside		2	[89]
47	quercetin-7-O-β-D-glucopyranoside		2	[89]
48	isorhamnetin-7-O-β-D-glucopyranoside		2	[89]
49	kaempferol-7-O-β-D-glucopyranoside		2	[89]
50	strophanthidin	Cardiotonic glycoside compounds	1	[93]
51	evomonoside		1, 2	[93,94]
52	helveticoside		1	[93]
53	evobioside		1	[93]
54	erysimoside		1	[93]
55	descurainolide A	Lignans compounds	1	[95]
56	descurainolide B		1	[95]
57	syringaresinol		1	[95]
58	scopoletine	Coumarin compounds	1	[95]
59	isoscopoline		1	[96]
60	xanthtoxol		1	[96]
61	xanthtoxin		1	[96]
62	psoralene		1	[96]
63	bergaptane		1	[96]
64	3,4,5-trimethoxycinnamic acid (4-methoxy sinapic acid)	Organic acid compounds	1	[88]
65	sinapic acid ethyl ester		1	[88]
66	descuraic acid		1	[97]
67	isovanillic acid		1	[98]
68	syringic acid		1	[98]
69	p-hydroxybenzoic acid		1	[98]
70	p-hydroxybenzaldehyde		1	[98]
71	nicotinic acid		1	[98]
72	sinapic acid		1	[87]
73	descurainoside B	Benzopyrone compounds	1	[99]
74	3,5-dimethoxy-4-hydroxybenzaldehyde (syringaldehyde)	Phenolic compounds	1	[87]
75	4-pentenamide	Unsaturated fatty amide	1	[99]
76	β-amyrin	Pentacyclic triterpenoids	1	[96]
77	β-sitosterol	Steroid compounds	1, 2	[87,89]
78	cholesterol		1	[96]
79	β-daucosterol (or β-sitosterol-3-O-β-D-glucoside)		1, 2	[87,89]
80	sinapine bisulfate	Benzodiazepine compounds	1	[87]
81	uracil		1	[99]
82	thymine		1	[99]
83	scopoline		1	[96]
84	3-methoxyinositol	Others	1	[99]
85	5-hydroxymethylfurfural		1, 2	[90,91,99]
86	2,5-dimethyl-7-hydroxychromone		1	[99]
87	descurainin		1	[95]

Note: Source 1 (*Descurainiae semen*); Source 2 (*Lepidii semen*). TCMSP (Traditional Chinese Medicine Systems Pharmacology Database and Analysis Platform) is a comprehensive database for herbal medicine research.

## 4. The Sulfur Compounds Found in *Descurainiae Semen* and *Lepidii Semen*

**Figure 5:**
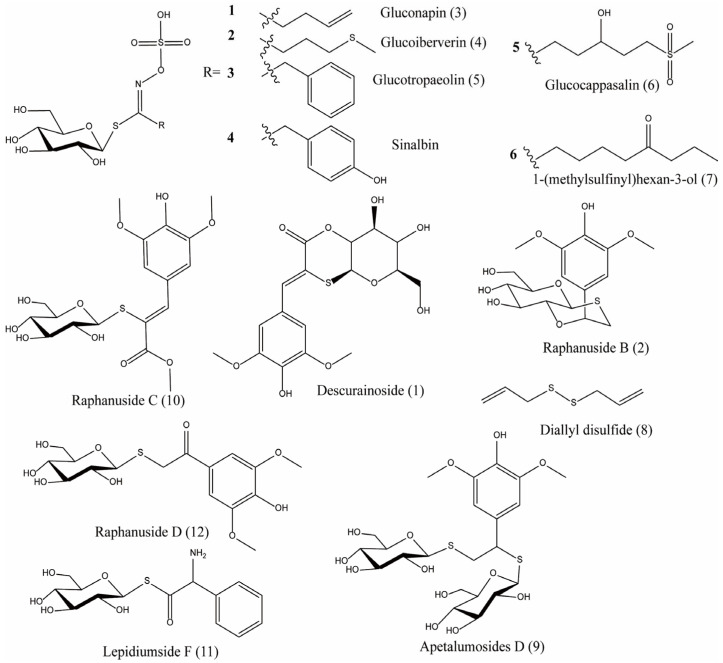
The sulfur compounds in *Descurainiae semen*.

**Figure 6:**
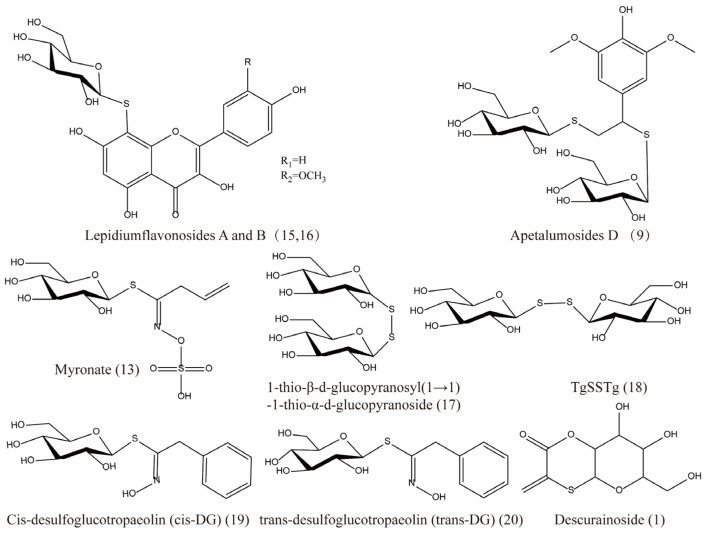
The sulfur compounds in *Lepidii semen*.

**Figure 7:**
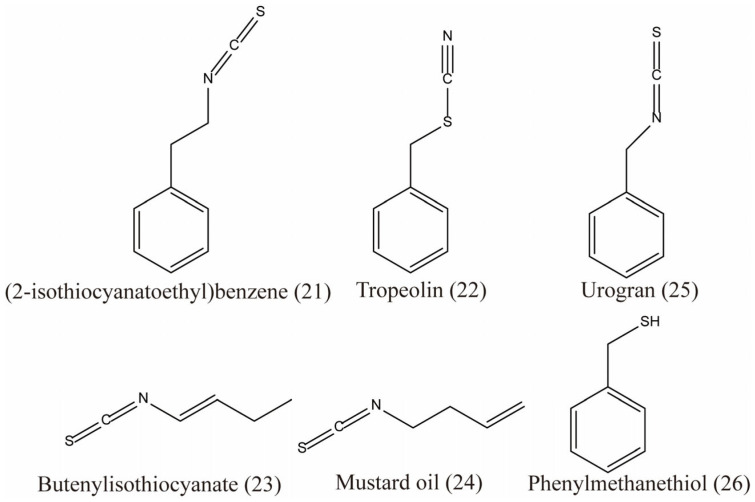
The sulfur compounds in TCMSP platform for *Lepidii semen* and *Descurainiae semen*.

The sulfur compounds presented in Figure 5, Figure 6 and Figure 7 (drawn using ChemBioDraw Ultra, https://chembiodraw-ultra.software.informer.com/) were systematically curated from existing phytochemical and pharmacological literature, as referenced for each compound. In the original studies cited, the identification and characterization of these compounds in *Lepidii semen* and *Descurainiae semen* were consistently established through a standard series of analytical techniques. These methods primarily involved chromatographic separation, most commonly high-performance liquid chromatography (HPLC) or thin-layer chromatography (TLC), coupled with structural elucidation by mass spectrometry (MS) and/or nuclear magnetic resonance (NMR) spectroscopy. The consistent application of these robust and widely accepted techniques across the primary literature provides a high degree of confidence in the compound assignments reported herein.

The sulfur compounds are as follows: descurainoside (1) [90,91], raphanuside B (2) [84], gluconapin (3) [84], glucoiberverin (4) [84], glucotropaeolin (5) [84], glucocappasalin (6) [84], 1-(methylsulfinyl)hexan-3-ol (7) [84], diallyl disulfide (8) [85], apetalumosides D (9) [47], raphanuside C (10) [83], lepidiumside F (11) [84], raphanuside D (12) [83], myronate (13) [85], sinalbin (14) [85], lepidiumflavonosides A and B (15, 16) [48], 1-thio-*β*-d-glucopyranosyl(1→1)-1-thio-*α*-d-glucopyranoside (17) [47], TgSSTg (18) [47], cis-desulfoglucotropaeolin (cis-DG) (19) [49], trans-desulfoglucotropaeolin (trans-DG) (20) [49], (2-isothiocyanatoethyl)benzene (21), tropeolin (22), butenylisothiocyanate (23), mustard oil (24), urogran (25) and phenylmethanethiol (26) (Table S1 in Supplementary Materials).

## 5. Theoretical Insights into Sulfur Compounds Targeting the PI3K/AKT Pathway

Molecular docking is a widely employed method in drug discovery and design, serving as a computational technique to investigate the interaction and recognition between receptors and ligands. It is a theoretical simulation approach used to study intermolecular interactions and predict binding modes and affinities [100]. In this paper, molecular docking methods were utilized to evaluate whether sulfur compounds from *Lepidii semen* and *Descurainiae semen* can bind with PI3K/AKT proteins (8TU6/7NH5). The procedure was conducted as part of the Supplementary Materials. The docking results indicated that most sulfur compounds from these seeds could potentially bind to the active sites of PI3K and Akt with favorable docking energies. Among them, glucotropaeolin, gluconapin, glucoiberverin, glucocappasalin, tropeolin, I-(methylsulfonyl)hexan-3-ol, glucoiberverin, lepidium-flavonosides A, raphanuside B, lepidium-flavonosides B, raphanuside D, (2-isothiocyanatoethyl)benzene, gluconapin, glucotropaeolin, glucocappasalin, raphanuside, descurainoside, tropeolin, lepidiumside F and urogran demonstrate greater potential (Figures S1 and S2 in Supplementary Materials). These computational observations hypothesize that the radioprotective efficacy of the seeds may be partly mediated through the modulation of the PI3K/AKT pathway by their inherent sulfur compounds. It is important to emphasize that these docking results provide a preliminary theoretical foundation and should be interpreted as a hypothesis-generating tool.

The glucosinolates found in Lepidii Semen and Descurainiae Semen and their enzymatically generated isothiocyanate derivatives are recognized as the principal bioactive compounds underlying the plant’s antioxidant, anti-inflammatory, and their link to the PI3K/Akt Signaling Pathway [42,78,79,80,81]. The potent antioxidant and anti-inflammatory properties previously attributed to these sulfur compounds align with the potential activation of cytoprotective signaling pathways like PI3K/AKT. Nevertheless, these in silico predictions require further validation through targeted experimental studies, such as in vitro binding assays and functional pathway analyses, to conclusively establish the mechanism of action. This integrative approach of linking computational predictions with documented pharmacological activities highlights a promising direction for future research into natural radioprotective agents.

## 6. Summary and Perspectives

Cruciferous plants are recognized as a highly radiation-resistant species, a property intrinsically linked to their rich content of natural radioprotective substances, particularly sulfur-containing compounds [101,102]. These cruciferous-derived compounds, known for their potent antioxidant and anti-inflammatory properties, can mitigate radiation-induced damage by attenuating oxidative stress and inflammatory responses [103,104]. While sulfur-containing compounds are a cornerstone in the design of synthetic anti-radiation drugs [105], the radioprotective potential of their natural counterparts has not been systematically investigated. Given that cruciferous plants are premium sources of diverse sulfur compounds [106], they represent a promising frontier for discovering novel radioprotective agents and expanding the known biological activities of these phytochemicals.

A pivotal pathway in the cellular response to radiation is PI3K/AKT. Its activation enhances cellular resistance to ionizing radiation [107] by integrally regulating processes of oxidative stress, inflammation, and apoptosis [108]. The PI3K/AKT pathway is a critical mediator of cell survival following radiation injury, acting by modulating the associated damaging processes [50].

(1) Antioxidant Mechanisms: Specifically, a key mode of action for many pharmacological and natural radioprotective agents is the activation of the PI3K/AKT signaling pathway. Once activated, this kinase cascade leads to the phosphorylation and nuclear translocation of the transcription factor Nrf2, a master regulator of the cellular antioxidant response. This results in the upregulated expression of heme oxygenase-1 (HO-1) and a suite of other cytoprotective enzymes. Through this coordinated Nrf2/HO-1 axis, these agents effectively scavenge and neutralize radiation-induced reactive oxygen species (ROS), thereby reducing intracellular ROS accumulation, preventing oxidative damage to lipids, proteins, and DNA, and enhancing cell survival post-irradiation [57,58]. For example, one of the core pharmacological effects of amifostine is its effective inhibition of oxidative stress. It achieves protection of normal tissues through two mechanisms: directly eliminating free radicals and indirectly enhancing the endogenous antioxidant enzyme system within cells [109].
(2) Anti-inflammatory Pathways: Beyond its antioxidant role, amifostine potently mediates anti-inflammatory cytoprotection by suppressing dysregulated inflammatory signaling. A primary mechanism involves its active metabolite, amifostine, directly inhibiting the activation of the PI3K/AKT pathway in normal cells. This inhibition blocks the subsequent phosphorylation and nuclear translocation of the pro-inflammatory master transcription factor NF-κB. Consequently, the expression of its downstream target genes is significantly downregulated, leading to a substantial reduction in the synthesis and release of key pro-inflammatory cytokines and enzymes, including TNF-α, IL-1β, IL-6, and COX-2. This systematic dampening of the PI3K/AKT/NF-κB axis attenuates radiation- or chemotherapy-induced inflammatory cascades, thereby preventing secondary tissue damage and contributing to the preservation of normal tissue architecture and function [110].
(3) Anti-apoptotic Effects: Furthermore, amifostine directly promotes cell survival by executing robust anti-apoptotic effects through the regulation of the PI3K/AKT/mTOR axis. Its active thiol metabolite, amifostine, facilitates the activation of PI3K, leading to the phosphorylation and activation of AKT at key serine/threonine residues. Activated AKT serves as a central node, directly phosphorylating and inactivating critical pro-apoptotic factors such as BAD and procaspase-9, thereby blocking the intrinsic mitochondrial apoptosis pathway. Concurrently, AKT stimulates the mTOR complex, a master regulator of cell growth and metabolism, which promotes protein synthesis and inhibits autophagy-associated cell death. This concerted signaling cascade, initiated by amifostine, shifts the cellular balance decisively towards survival, enhancing the resistance of normal tissues to the genotoxic and cytotoxic insults of radiotherapy and chemotherapy [60,61,110].

However, the PI3K/AKT signaling pathway exhibits a critical and complex dualism in medical science. In oncology, its hyperactivation is a well-established driver of tumorigenesis, promoting cancer cell survival, proliferation, and resistance to therapy, making it a prime therapeutic target for inhibition. Conversely, in the context of normal tissue radioprotection, the same pathway is essential for cellular defense, where its activation can protect healthy cells from radiation-induced damage and apoptosis [110,111]. The scope of this review is exclusively on radioprotection of normal tissues.

Integrating Theoretical Predictions and Future Directions: To theoretically explore the potential of *Descurainiae semen* and *Lepidii semen*, we conducted a molecular docking analysis. The results indicated that sulfur compounds from these seeds show relatively favorable binding energies to PI3K/AKT, suggesting a plausible molecular mechanism for their purported radioprotective effects. However, it is imperative to state that these are in silico predictions. Their primary value lies in generating a robust and testable hypothesis, not in serving as conclusive evidence. Therefore, the central proposition of this review—that the sulfur compounds in these seeds confer radioprotection via PI3K/AKT activation—requires rigorous experimental validation. We propose a concerted research agenda to bridge this gap. (1) Targeted Compound Screening: Prioritize the sulfur compounds identified in this review for in vitro testing in irradiated cell models. Key endpoints should include clonogenic survival, DNA damage repair, and direct measurement of PI3K/AKT pathway activation via Western blot. (2) In Vivo Efficacy Studies: Evaluate the most promising candidates from in vitro work in animal models of radiation injury, assessing critical outcomes such as 30-day survival, hematopoietic recovery, and gastrointestinal integrity. (3) Mechanistic Confirmation: Employ techniques like Surface Plasmon Resonance (SPR) to experimentally verify the physical binding between the compounds and the PI3K/AKT proteins, providing direct biochemical support for the docking predictions.

From In Silico Prediction to Practical Application: To directly address the path for eventual testing and application, we propose a clear translational strategy. The journey from computational prediction to a viable radioprotective agent involves critical decisions that our review helps to inform. (1) Lead Compound Selection: The docking studies prioritize specific sulfur compounds from the chemical libraries of *Lepidii semen* and *Descurainiae semen* for immediate experimental follow-up. The next step is not further docking, but procuring these prioritized compounds as purified standards for in vitro and in vivo validation. (2) Defining the Therapeutic Regimen: Future preclinical studies must explicitly determine the optimal dosing schedule (prophylactic vs. therapeutic administration), route of administration, and whether a standardized seed extract or isolated pure compounds offer the best efficacy and safety profile. This will define the practical use case for these agents. (3) Critical Safety and Advantage Assessment: A pivotal question to be answered is whether these natural sulfur compounds possess a superior therapeutic index compared to the synthetic agents listed in the introduction. Comparative toxicity studies are essential to validate the hypothesis that natural derivatives may offer reduced side effects, which is a primary motivation for their investigation. This roadmap outlines a direct path to translate the hypotheses generated herein into tangible candidates, moving beyond computational prediction towards defining their eventual clinical utility, dosing, and safety advantages.

The biological activities of sulfur compounds from various cruciferous plants have been primarily focused on anti-tumor, antibacterial, and anti-inflammatory effects. For instance, raphanin exhibits significant anti-tumor and anti-inflammatory properties [112], and Maca-derived compounds show neuroprotective effects [113]. However, the anti-radiation activity of isolated sulfur compounds remains a markedly underexplored field. By synthesizing the existing knowledge and proposing a clear, mechanistic hypothesis for *Lepidii semen* and *Descurainiae semen*, this review aims to open a new avenue for research into cruciferous sulfur compounds, ultimately laying the groundwork for developing novel, effective, and natural radioprotective drugs.

**Figure 8:**
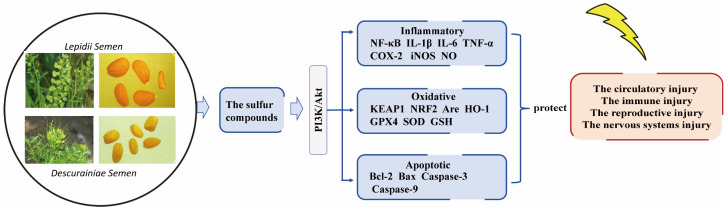
Proposed molecular mechanisms underlying the radioresistance of *Lepidium apetalum* and *descurainia sophia* seeds.

In conclusion, cruciferous plants are rich in natural sulfur-containing compounds with antioxidant and anti-inflammatory activities, making them an ideal source for developing new radiation protection agents. The underlying mechanism may involve activating the PI3K/Akt pathway to coordinately regulate oxidative stress, inflammation, and apoptosis (as shown in Figure 8). Although molecular docking predicted the binding potential of the components in *Lepidii semen* and *Descurainiae semen* to this pathway, this hypothesis needs to be verified through systematic experiments, such as targeted compound screening, in vitro and in vivo validation; confirmation of the binding mechanism is needed to fill the research gap in this field.

## Figures and Tables

**Table 1:** Compounds with anti-radiation activity in sulfur-containing compounds.

Species	Compounds
Sulfhydryl	L(+)-Cysteine [8], Cysteamine [9], Unithiol [10]. Amifostine thiol [11], Dithiothreitol [12], Amifostine [13], Glutathione [14]
Disulfide	Cystamine dihydrochloride [15], 5-(1,2-dithiolan-3-yl) pentanoic acid [16]
Organic-thiosulfate	S-(2-aminoethyl) O-hydrogen sulfurothioate [17], S-(1-aminopropan-2-yl) O-hydrogen sulfurothioate [18], S-(2-aminoproply) O-hydrogen sulfurothioate [19], 2-(N-decylamino)ethanethiosulfuric acid [20], Phenylalkylaminoethyl thiosulfates [21], N-substituted S-2-aminoethylthiosulfates [22], S-(2-((5-((2-isopropyl-5-methylcyclohexyl)oxy)pentyl)amino)ethyl) O-hydrogen sulfurothioate [23], S-(2-((5-((5-methyl-2-(1-methylethyl)cyclohexyl)oxy) [24], S-(2-(guanidinoethyl) O-hydrogen sulfurothioate [25], sodium cysteine thiosulfonate [26], Cystaphos [27], S-(2-((2-(methylamino)ethyl)amino)ethyl) O, O-dihydrogen phosphorothioate and Lithium S-(1-(dimethylamino)propan-2-yl)phosphorothioate [28,29]
Tetrahydrothiazole	2,2-dimethylthiazolidine [30], 3-(5-(pentylthio)pentyl)thiazolidine [31]
Thiourea derivatives	(2-Aminoethyl)isothiourea hydrobromide [32], Carbamimidothioic acid, 2-aminopropyl ester, dihydrobromide [33]
Sulfone and sulfoxide compounds	Dimethyl sulfoxide [34,35], Ex-RAD [36]

## Data Availability

No new data were created or analyzed in this study. Data sharing is not applicable to this article.

## Supplementary Materials

The following supporting information can be downloaded at https://www.mdpi.com/article/10.3390/ijms27041847/s1. References [13,42,110,114,115,116,117,118,119,120] are cited in Supplementary Materials.

## Abbreviations

Akt	Protein Kinase B
PI3K	Phosphatidylinositol 3-Kinase
CNKI	China National Knowledge Infrastructure
IL-1β	Interleukin-1 Beta
IL-6	Interleukin-6
NO	Nitric Oxide
iNOS	Inducible Nitric Oxide Synthase
HO-1	Heme Oxygenase-1
COX-2	Cyclooxygenase-2
IL-10	Interleukin-10
NF-κB	Nuclear Factor Kappa-Light-Chain-Enhancer of Activated B Cells
NMR	Nuclear Magnetic Resonance
PDB	Protein Data Bank
PDBQT	Protein Data Bank, Partial Charge (Q), and Atom Type (T)
ROS	Reactive Oxygen Species
TCM	Traditional Chinese Medicine
TCMSP	Traditional Chinese Medicine Systems Pharmacology
TNF-*α*	Tumor Necrosis Factor-Alpha
mTOR	Mammalian Target of Rapamycin
