# Supplementary material for: From the Literature on Mining to Computational Verification: A Review of the Anti-Radiation Mechanisms of Sulfur Compounds in the Seeds of Lepidium apetalum Willd and Descurainia sophia (L.) Webb ex Prantl
Source: Int J Mol Sci. 2026 Feb 14;27(4):1847. doi: 10.3390/ijms27041847 (PMC12940398; doi:10.3390/ijms27041847)
Supplement: Supplementary file 1 [file ijms-27-01847-s001.zip › ijms-4087637-supplementary.pdf]

SUPPLEMENTARY MATERIAL

The sulfur compounds found in *Lepidii Semen* and *Descurainiae Semen* were primarily retrieved from specialized databases, including TCMSP (https://www.tcmsp-e.com/), PubMed (https://pubmed.ncbi.nlm.nih.gov/), and SciFinder (https://scifinder.cas.org/). These platforms were queried using the seed names ("*Lepidii Semen*", "*Lepidium apetalum*", "*Descurainiae Semen*", "*Descurainia sophia*") as primary keywords to identify their known chemical constituents（table 2）, with a focus on sulfur-containing compounds.

Table S1 Sulfur-containing compounds *of Descurainiae Semen and Lepidii Semen*

No.	Chemical Composition	Classification of Compounds	Plant Source	evidence tier
1	descurainoside		1	in silico only
2	raphanuside B		1	in silico only
3	gluconapin		1	in silico only
4	glucoiberverin		1	in silico only
5	glucotropaeolin		1	in silico only
6	glucocappasalin		1	in silico only
7	1-(methylsulfinyl)hexan-3-ol		1	in silico only
8	diallyl disulfide		1	in silico only
9	apetalumosides D		1	in silico only
10	raphanuside C		1	in silico only
11	lepidiumside F		1	in silico only
12	raphanuside D		1	in silico only
13	myronate	Organic sulfur compounds	2	in silico only
14	sinalbin		1	in silico only
15	lepidiumflavonosides A		2	in silico only
16	lepidiumflavonosides B		2	in silico only
17	1-thio-β-d-glucopyranosyl(1→1)-1-thio-α-d-glucopyranoside		2	in silico only
18	TgSSTg		2	in silico only
19	cis-desulfoglucotropaeolin (cis-DG)		2	in silico only
20	trans-desulfoglucotropaeolin (trans-DG)		2	in silico only
21	(2-isothiocyanatoethyl)benzene		1, 2	in silico only
22	tropeolin		1, 2	in silico only
23	butenylisothiocyanate		1, 2	in silico only
24	mustard oil		1, 2	in silico only

25	urogran	1, 2	in silico only
26	phenylmethanethiol	1, 2	in silico only

Note: Source 1 (*Descurainiae Semen*); Source 2 (*Lepidii Semen*). 1-26 is the organic sulfur compound extracted from *Descurainiae Semen* and *Lepidii Semen.*

Molecular docking methods:

1) Protein receptor file preparation: Based on the previous literature research results, the 3D structure of the target protein was retrieved from the protein database (PDB) (http://www. rcsb.org/) [1] and the PDB format file was downloaded. The protein was then configured in AutoDock 4.2.6 and AutoDockTools 1.5.7 program [2] as follows: Remove the water, replace it with hydrogen, designate the protein as a receptor, and save the structure as a PDBQT protein receptor file.

2) Ligand file preparation: Drug molecule structures were obtained from TCMSP (https://www.tcmsp-e.com/#/database) and PubChem (https://pubchem.ncbi.nlm. nih.gov/) databases [3]. Similarly, in AutoDock 4.2.6 and AutoDockTools 1.5.7 program, we set the drug as follows: delete water, add hydrogen, and set the drug as ligand. The torsion tree was automatically configured in the software. Exported to a ligand file in PDBQT format.

3) Definition of docking parameters: Import the receptor and ligand PDBQT structures into AutoDock 4.2.6 and AutoDockTools 1.5.7 program and the molecular docking range was defined. The grid center was set at the geometric center of the target protein, and the center coordinate (center x/y/z) and box size (size x/y/z) parameters were adjusted to ensure complete coverage of the protein by the docking box [4].

Molecular docking and visualization: Molecular docking was performed in AutoDock 4.2.6 and AutoDockTools 1.5.7 by detecting protein macromolecules, inserting small drug molecules, configuring operational methods, and setting docking parameters. A total of 50 independent global docking runs were performed to ensure conformational sampling robustness. Binding poses were ranked by predicted binding energy (kcal/mol), with the lowest-energy pose selected as the most favorable binding conformation. Docking results were saved in PDBQT format, and the top-scoring protein-ligand complex was converted to PDB format for structural visualization using PyMOL (version 4.6.0)

4) Molecular Docking Validation and Benchmarking:

To ensure the reliability and robustness of our molecular docking predictions, a comprehensive validation protocol was implemented prior to screening the sulfur compounds. (1) Docking Protocol Validation. The accuracy of our docking procedure was verified by performing a re-docking experiment. The ligand (e.g., Amifostine) was the first radiation protection agent approved by the FDA

[5,6]. Studies have shown that amifostine can alleviate oxidative stress, inflammation and apoptosis by inhibiting the activation of the PI3K/Akt/ mTOR signaling pathway [7]). Quercetin is a natural flavonoid compound widely present in plants, which has multiple biological activities such as antioxidation, anti-inflammation and anti-cancer [8]. Quercetin has a significant inhibitory effect on the PI3K/AKT signaling pathway[9] . The protein kinase Akt plays a pivotal role in cellular processes [10]. We selected amifostine and quercetin to perform three molecular docking with Akt (PDB ID:7NH5) and calculated the RMSD value. This ligand was then re-docked into the original binding site using the same parameters applied in our study. The structural similarity between the computationally re-docked pose and the original crystal structure pose was quantified by calculating the root-mean-square deviation (RMSD). An RMSD value was obtained, which is below the widely accepted threshold of 2.0 Å, confirming that our docking protocol can faithfully reproduce experimentally observed binding modes. (2)Benchmarking Against Known Agents. To contextualize the docking scores of the sulfur compounds from Lepidii Semen or Descurainiae Semen, we docked a known radioprotective agent and a reference inhibitor of the PI3K/Akt pathway under identical conditions. The reference compound (Amifostine) yielded a docking score of -6.50 kcal/mol. Several of our natural sulfur compounds demonstrated comparable or even more favorable (i.e., more negative) docking energies than this reference, suggesting their potential as high-affinity ligands. This comparison provides a benchmark for assessing the predicted binding strength of the investigated compounds. (3)Criteria for Interpreting Binding Affinity. Based on the distribution of docking scores from our library and established conventions in the literature, we defined the following criteria to classify the predicted binding affinity: Strong interaction: Docking energy < -1.2 kcal/mol; Moderate interaction: Docking energy < 0 kcal/mol; Weak interaction: Docking energy > 0 kcal/mol.

Hypothesis-Generating Docking Analysis: the purpose is to provide a prioritization for

subsequent experimental verification, rather than to provide conclusive results.

Compound No.	Compounds	PI3K	The binding site	Binding energy
1	Descurainoside	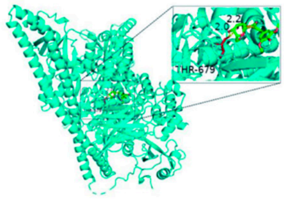	THR-679	-4.67
2	Raphanuside B	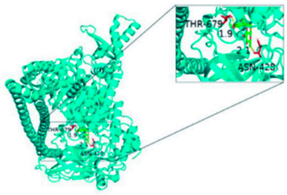	THR-679, ASN-428	-4.08
3	Gluconapin	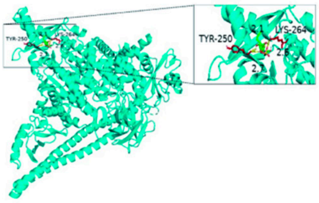	TYR-250, LYS-264	-6.23
4	Glucoiberverin	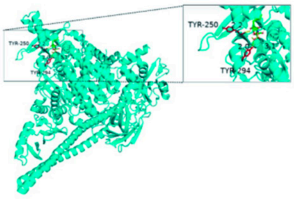	TYR-250, TYR-294	-6.11
5	Glucotropaeolin	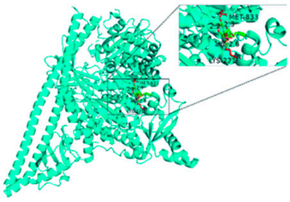	MET-833, LYS-271	-6.66
6	Glucocappasalin	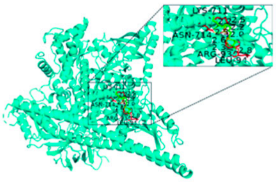	LYS-711, ASN-714, ARG-93, LEU-94	-5.08

A

Compound No.	Compounds	PI3K	The binding site	Binding energy
7	1-(methylsulfonyl) hexan-3-ol	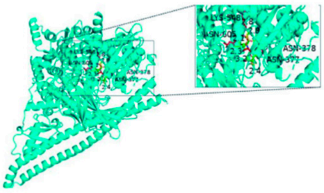	LYS-548, ASN-377, ASN-378, ASN-605	-5.14
8	diallyldisulfane	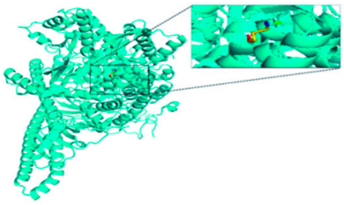	—	-3.55
9	Apetalumosides D	—	—	1.21
10	Raphanuside C	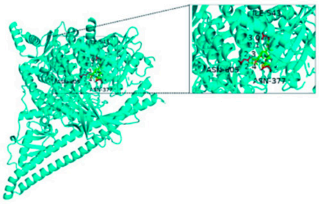	ASN-377, ASN-605, ILE-543	-3.81
11	Lepidiumside F	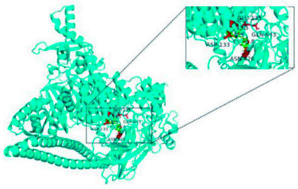	GLU-135, GLN-643, ASP-133, ASN-426	-3.92
12	Raphanuside D	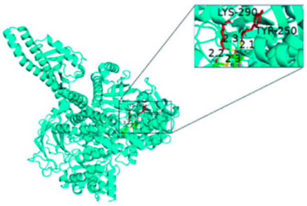	LYS-290, TYR-250	-2.43
13	Myronate	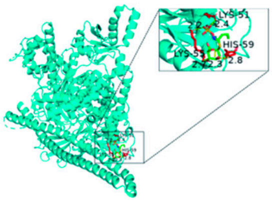	LYS-51, LYS-55, HIS-59	-1.47

B

Compound No.	Compounds	PI3K	The binding site	Binding energy
14	Sinalbin	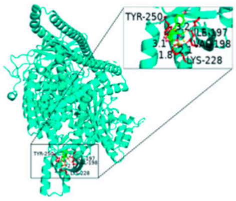	TYR-250, ILE-197, VAL-198, LYS-228	-2.68
15	Lepidiumflavonosides A	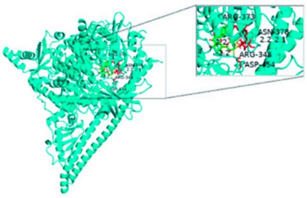	ARG-373, ASN-378, ARG-348, ASP-454	-2.72
16	Lepidiumflavonosides B	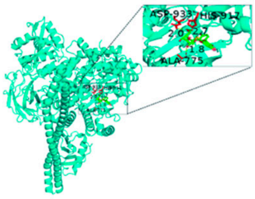	ASP-933, HIS-917, ALA-775	-2.23
17	1-thio-β-D-glucopyranosyl (1→1)-1-thio-α-D-glucopyranoside	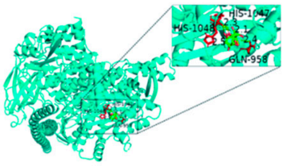	HIS-1047, HIS-1048, GLN-958	-0.19
19	Cis-desulfoglucotropaeolin	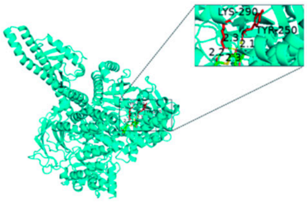	VAL-461, GLY-1007, ASN-457	-3.64
20	Trans-desulfoglucotropaeolin	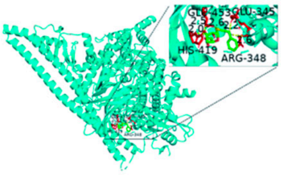	GLU-453, GLU-345， HIS-419， ARG-348	-2.88

C

Compound No.	Compounds	PI3K	The binding site	Binding energy
21	(2-isothiocyanatoethyl) benzene	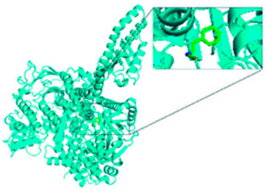	—	-4.75
22	Tropeolin	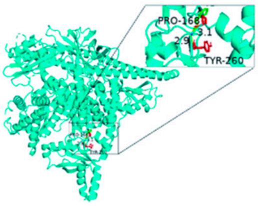	PRO-168, TYR-260	-5.23
23	Butenylisothiocyanate	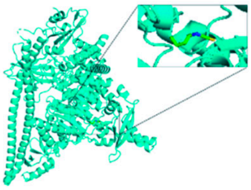	—	-3.4
24	Mustard oil	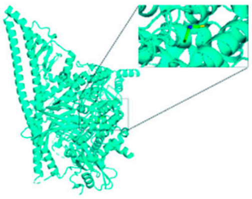	—	-3.6
25	Urogran	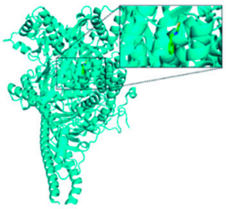	—	-4.79
26	Phenylmethanethiol	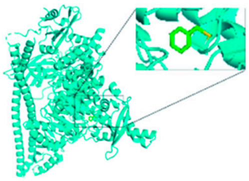	—	-4.4

D

Figure S1(A-D). Molecular docking results of organic sulfur compounds with PI3K (PDB ID:8TU6)

Note: The lowest-energy conformation is identified as the thermodynamically most favorable binding pose.

Compound No.	Compounds	AKT	The binding site	Binding energy
1	Descurainoside	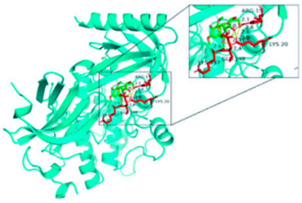	ARG-15, LYS-20, GLU-298, LYS-297	-6.82
2	Raphanuside B	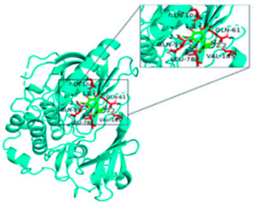	GLN-61, GLN-59, GLN-104, VAL-185, LEU-78	-6.31
3	Gluconapin	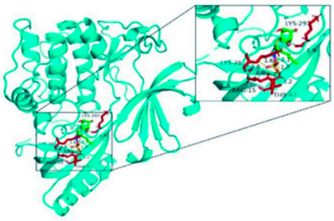	LYS-297, LYS-20, ARG-15, THR-87	-10.39
4	Glucoiberverin	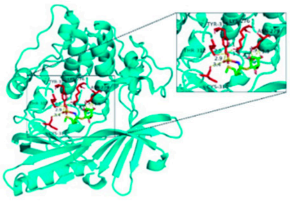	TYR-315, LYS-276, ASN-279, THR-312, CYS-310	-6.75
5	Glucotropaeolin	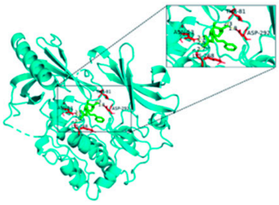	THR-81, ASN-53, ASP-292, LYS-268	-9.64
6	Glucocappasalin	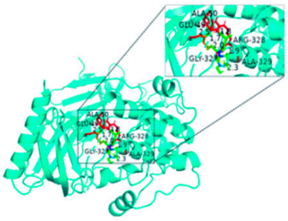	ALA-50, GLU-49, ARG-328, GLY-327, ALA-329	-8.91

A

Compound No.	Compounds	AKT	The binding site	Binding energy
7	1-(methylsulfonyl) hexan-3-ol	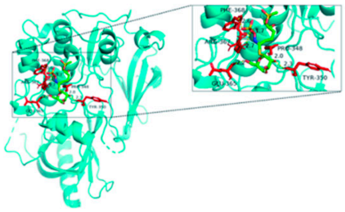	PHE-368, ARG-367, PRO-348, GLU-365, TYR-350	-8.76
8	diallyldisulfane	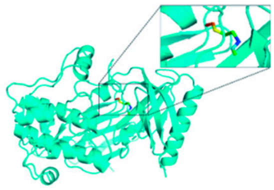	—	-4.22
9	Apetalumosides D	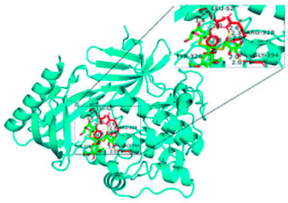	LEU-52, ARG-328, TYR-326, GLY-394	-3.02
10	Raphanuside C	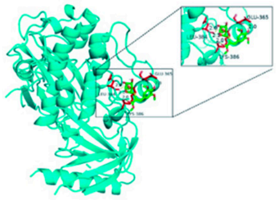	GLU-365, LEU-384, LYS-386	-4.35
11	Lepidiumside F	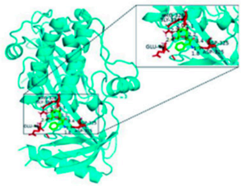	ARG-328, GLY-394, GLU-49, ASP-325	-5.87
12	Raphanuside D	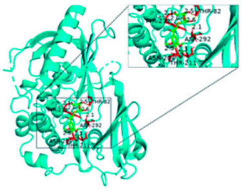	THR-82, TYR-272, ASP-292, ASN-204, THR-211	-6.01

B

Compound No.	Compounds	AKT	The binding site	Binding energy
13	Myronate	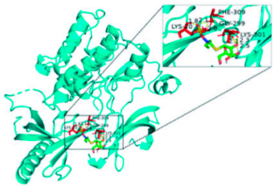	PHE-309, LYS-20, GLY-299, LYS-301	-3.66
14	Sinalbin	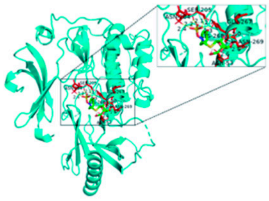	SER-205, ASN-204, GLU-267, LYS-268, ASN-53, ASN-269	-4.54
15	Lepidium-flavonosides A	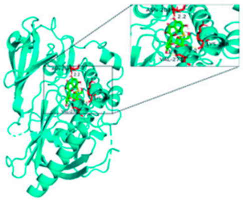	ASN-204, LYS-268, VAL-271	-6.73
16	Lepidium -flavonosides B	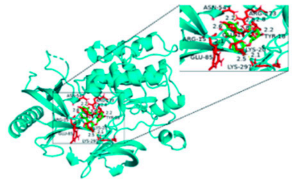	ASN-54, ARG-273, GLU-17, TYR-18, ARG-15, LYS-20, GLU-85, LYS-297	-6.24
17	1-thio-β-d-glucopyranosyl (1→1)-1-thio-β-d-glucopyranosid	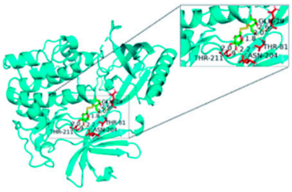	GLN-79, THR-81, THR-211, ASN-204	-4.86
19	Cis-desulfo glucotropaeolin	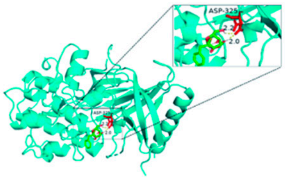	ASP-325	-4.62

C

Compound No.	Compounds	AKT	The binding site	Binding energy
20	Trans-desulfo glucotropaeolin	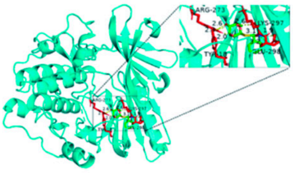	ARG-273, LYS-297, TYR-18, GLU-298	-4.86
21	(2-isothiocyanatoethyl) benzene	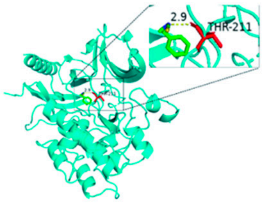	THR-211	-5.93
22	Tropeolin	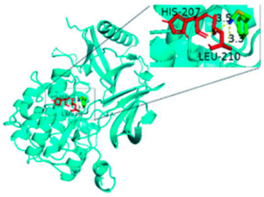	HIS-207, LEU-210	-5.87
23	Butenylisothiocyanate	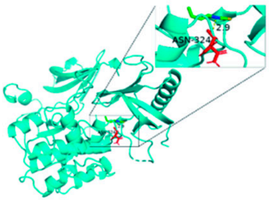	ASN-324	-4.28
24	Mustard oil	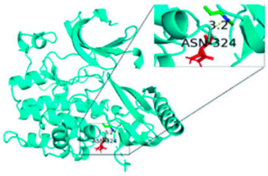	ASN-324	-4.13
25	Urogran	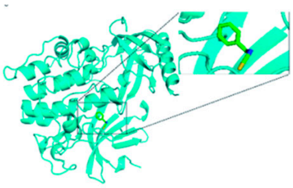	–	-5.46
26	Phenylmethanethiol	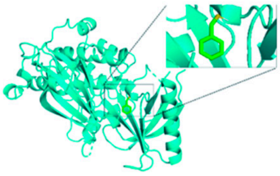	–	-4.85

D

Figure S2(A-D). Molecular docking results of organic sulfur compounds with Akt (PDB ID:7NH5)

Note: The lowest-energy conformation is identified as the thermodynamically most favorable binding pose.

References

1. Burley, S.K., Bhikadiya, C., Bi, C. Bittrich, S., Chen, L., Crichlow, G.V., Christie, C.H., Dalenberg, K., Di Costanzo, L., Duarte, J.M., Dutta, S., Feng, Z., Ganesan, S., Goodsell, D.S., Ghosh, S., Green, R.K., Guranovi´c, V., Guzenko, D., Hudson, B.P. ,Lawson, C.L., Liang, Y., Lowe, R., Namkoong, H., Peisach, E., Persikova, I., Randle, C., Rose, A., Rose, Y., Sali, A., Segura, J., Sekharan, M., Shao, C., Tao, Y.P., Voigt, M., Westbrook, J.D., Young, J.Y., Zardecki, C., Zhuravleva, M. RCSB Protein Data Bank: powerful new tools for exploring 3D structures of biological macromolecules for basic and applied research and education in fundamental biology, biomedicine, biotechnology, bioengineering and energy sciences. Nucleic Acids Res. 49: 437–451, 2021.
2. Seeliger, D., de Groot, B.L. Ligand docking and binding site analysis with PyMOL and Autodock/Vina. J Comput Aided Mol Des. 5:417-22, 2010.
3. Kim, S.B., Seo, Y.S., Kim, H.S., Lee, A.Y., Chun, J.M., Moon, B.C., Kwon, B.I. Anti-asthmatic effects of lepidii seu Descurainiae Semen plant species in ovalbumin-induced asthmatic mice. J Ethnopharmacol. 15: 112083, 2019.
4. He Q, Liu C, Wang X, Rong K, Zhu M, Duan L, Zheng P, Mi Y. Exploring the mechanism of curcumin in the treatment of colon cancer based on network pharmacology and molecular docking. Front Pharmacol. 2023 Feb 15;14:1102581.
5. Ji, L., Cui, P., Zhou, S., Qiu, L., Huang, H., Wang, C., Wang, J. Advances of Amifostine in Radiation Protection: Administration and Delivery. Mol Pharm. 20: 5383-5395, 2023.
6. King, M., Joseph, S., Albert, A., Thomas, T.V., Nittala, M.R., Woods, W.C., Vijayakumar, S., Packianathan, S. Use of Amifostine for Cytoprotection during Radiation Therapy: A Review. Oncology. 2:61-80, 2020
7. Yang, W., Pan, L., Cheng, Y., Wu, X., Huang, S., Du, J., Zhu, H., Zhang, M., Zhang, Y. Amifostine attenuates bleomycin-induced pulmonary fibrosis in mice through inhibition of the PI3K/Akt/mTOR signaling pathway. Sci Rep. 13:10485, 2023.
8. Alizadeh S.R., Ebrahimzadeh M.A. Quercetin derivatives: Drug design, development, and biological activities, a review. Eur J Med Chem. 229:114068, 2022.
9. Liu F., Feng Q., Yang M., Yang Y., Nie J., Wang S. Quercetin prevented diabetic nephropathy by inhibiting renal tubular epithelial cell apoptosis via the PI3K/AKT pathway. Phytother Res. 7:3594-3606, 2024.
10. Uko N.E., Güner O.F., Matesic D.F., Bowen J.P. Akt Pathway Inhibitors. Curr Top Med Chem. 10:883-900, 2020.
